# Usefulness of Gastrojejunostomy Prior to Fundoplication in Severe Gastro-Esophageal Reflux Complicating Long-Gap Esophageal Atresia Repair: A Preliminary Study

**DOI:** 10.3390/children8010055

**Published:** 2021-01-17

**Authors:** Francesca Destro, Luciano Maestri, Milena Meroni, Federico Rebosio, Giulia Del Re, Cecilia Mantegazza, Valeria Calcaterra, Gloria Pelizzo

**Affiliations:** 1 Pediatric Surgery Department, “Vittore Buzzi” Children’s Hospital, 20154 Milan, Italy; francesca_destro@hotmail.com (F.D.); luciano.maestri@asst-fbf-sacco.it (L.M.); milena.meroni@asst-fbf-sacco.it (M.M.); f.rebosio@unibs.it (F.R.); giulia.delre@live.com (G.D.R.); 2 Department of Pediatrics, “Vittore Buzzi” Children’s Hospital, 20154 Milan, Italy; cecilia.mantegazza@asst-fbf-saccco.it (C.M.); valeria.calcaterra@unipv.it (V.C.); 3 Pediatric and Adolescent Unit, Department of Internal Medicine, University of Pavia, 27100 Pavia, Italy; 4 Department of Biomedical and Clinical Science “L. Sacco”, University of Milan, 20157 Milan, Italy

**Keywords:** esophageal atresia, malnutrition, growth, nutritional status, energy metabolism, feeding difficulty, dysphagia

## Abstract

Background: Gastro-esophageal reflux disease (GERD), requiring surgical correction, and nutritional problems are reported after long-gap esophageal atresia (LGEA) repair and might jeopardize the postoperative course in some babies. We report an exploratory evaluation of the role of transgastric jejunostomy (TGJ) as a temporary nutritional tool before surgery for GERD in LGEA. Methods: Seven infant patients operated on for LGEA with intra-thoracic gastro-esophageal junction (GEJ) and growth failure, requiring improvement in their nutritional profile in anticipation of surgery, were retrospectively evaluated. Post-surgical follow-up, including growth evolution, complications, and parental quality of life (QoL), were considered. Results: The TGJ was placed at a mean age of 8.6 ± 5.6 months. The procedure was uneventful and well-tolerated in all seven cases. At 6.6 ± 2.0 months after TGJ placement, significant weight gain (weight z-score −2.68 ± 0.8 vs −0.9 ± 0.2, *p* < 0.001) was recorded, allowing the GERD surgery to proceed. A significant difference in hospital admissions between 3 months before and post-TGJ insertion was noted (4.8 ± 0.75 vs. 1.6 ± 0.52, *p* < 0.01). A significant amelioration of QoL after TGJ placement was also recorded; in particular, the biggest improvements were related to parents’ perceptions of the general health and emotional state of their babies (*p* < 0.001). Conclusions: The placement of TGJ as a temporary nutritional tool in selected cases of LGEA could improve nutritional conditions and parental QoL before fundoplication, allowing successful surgical treatment of GERD to be carried out.

## 1. Introduction

Long-gap esophageal atresia (LGEA) occurs in 10% of patients with esophageal atresia [1,2], and represents a challenge for surgical issues, post-operative sequelae, and comorbidities.

It is still universally accepted that preserving the native esophagus is the best option for the patient [3,4,5,6]. However, delayed esophageal anastomosis with or without elongation is associated with a high rate of postoperative complications, such as gastroesophageal reflux disease (GERD).

The incidence of symptomatic GERD is reported in more than 50% of cases [2], and it requires aggressive treatment. Fundoplication is considered the most common major surgical procedure in patients with LGEA [1,7,8]. Nevertheless, the reduced length of the esophagus is often considered a limitation for surgery.

An additional problem relates to nutritional aspects after restoring the esophageal continuity [7]; as a consequence, patients with wide-gap LGEA often have feeding difficulties (feeding resistance, food refusal, difficulty in introducing solid food, selective feeding, long mealtimes) and nutritional impairment.

We report an exploratory evaluation on the role of transgastric jejunostomy (TGJ) as a temporary nutritional tool before surgical treatment of GERD in a case series of patients born with LGEA with an intra-thoracic gastro-esophageal junction (GEJ) and growth failure with the need to improve their nutritional profile in anticipation of surgery. Post-surgical follow-up, including growth evolution, complications, and parental quality of life (QoL), was considered.

## 2. Patients and Methods

### 2.1. Patients

In this retrospective study, seven infant patients (4 males and 3 females) operated on for LGEA (4 type A and 3 type C) were enrolled.

All infants presented clinical and radiological signs of severe GERD (vomiting, nutritional impairment, airway infections, and bodyweight < 3rd centile for sex and age according to WHO or Fenton growth charts in preterm infants [9]) despite aggressive medical treatment with proton pump inhibitors (omeprazole or lansoprazole 1–2 mg/kg/day), as well as positioning and feeding adjustments. The clinical features of the patients are reported in Table 1.

The study was performed in accordance with the Declaration of Helsinki principles and with the approval of the local Institutional Review Board (Fond. IRCCS Pol. S. Matteo, PV 20/11/2016 prot. 20160000069). After receiving information about the nature of the study, the patients’ parents or guardians gave written consent for their child’s participation.

### 2.2. Surgical Management

In all patients with LGEA (gap length > 3 vertebral bodies), a Stamm gastrostomy was performed at birth (with fistula closure when present) and esophageal anastomosis was carried out at a mean age of 4.6 months (age range 2.1–10.9 months), as reported in Table 2 and Table 3. The presence of LGEA was assumed at birth, evaluating the thoraco-abdominal x-ray, and later confirmed by a measurement performed inserting bougies in the upper (through the mouth) and lower (through the gastrostomy) pouches.

In selected cases with severe post-operative drug-resistant GERD and comorbidities, fundoplication was performed as the treatment of choice [3,4,5]. In all cases reported, “early” fundoplication was temporarily contraindicated due to severe malnutrition. The endoscopic placement of a jejunal tube was therefore positioned through the pre-existing gastrostomy before proceeding to surgery.

## 3. Methods

The study was designed in order to evaluate the nutritional role of TGJ prior to fundoplication by the analysis of three parameters (weight changes of growth course, hospital admissions, quality of life), as detailed below.

### 3.1. TGJ Insertion

The placement of the jejunal tube was performed in the operating room under general anesthesia with a 5 or 6 mm gastroscope inserted through the gastrostomy. When required, prior to endoscopy, progressive dilatation of the stoma was performed until the adequate caliber was reached. The gastroscope was directed through the pylorus, into the duodenum, and a guide-wire was passed up to the third duodenal portion. The endoscope was then retracted under direct vision and the jejunal tube was placed over the wire (MIC-KEY, Kimberley Clark, Draper, Utah, USA low-profile gastro-jejunostomy tube, diameter 16 Fr–length from 15 to 22 cm–depth from 1.7 to 2 cm). The insertion of the tube over the guidewire, which is nitinol covered with hydrophilic Teflon and is provided by a hydrophilic coated soft tip, 230 cm in length, is straightforward with proper lubrification of the tube (e.g., using paraffin oil). On the other hand, the removal of the guidewire can be tricky because there is the risk of curling and pulling back the tube as well. The maneuver should be performed cautiously: the tube is anchored to the gastric wall by balloon filling and it is kept in place with one hand while the guidewire is pulled out with the other hand, using constant pressure and avoiding jerks. A fluoroscopic evaluation at the end of the procedure was required to clear up any doubts on the correct positioning of the tube.

Approximately six months after gastro-jejunostomy placement, patients underwent Nissen fundoplication. At fundoplication, the gastro-jejunal tube was removed while the gastrostomy was maintained for about 1 month postoperatively in order to treat possible gas-bloating syndrome and dysphagia. Table 3 reports the clinical parameters of the patients at different surgical stages.

### 3.2. Nutritional Planning

Dietary schedules were tailored to every single patient following consultation with nutritional experts. The final planning included two bolus meals during the day and continuous night feeding with monomeric nutrient solutions. Meanwhile, oral stimulation and rehabilitation were strongly supported. Gastric venting from the gastric probe was employed at least two to three times per day to empty the stomach. After surgery, oral feeding was gradually introduced with good tolerance.

Patients were closely monitored during follow-up at our outpatient clinic until the nutritional goals were reached (usually 10–15 days), and then check-ups were arranged on the basis of individual needs.

### 3.3. Post-Surgical Monitoring

As post-surgical parameters, we analyzed:

(1) weight increases after the placement of the jejunal tube, at the time of fundoplication surgery. Bodyweight was measured with a beam scale and it was used as the most sensitive parameter to detect short-term weight variations;
(2) the number of hospital admissions during a period ranging from 3 months before to 3 months after TGJ positioning;
(3) parental quality of life (QoL) before and after TGJ positioning (by administration of Short-Form 36 questionnaire version 1.6 to parents before fundoplication). The questionnaire is a common patient-reported survey to assess the patients’ health. It is based on the evaluation of eight items: physical activity, role and physical activity, physical pain, general health, vitality, social activities, emotional state and mental health. Each item is associated with a score with a maximum value of 100; the lower the score, the more severe the disability.

In all the patients, a multidisciplinary approach with a dedicated team, including an otolaryngologist expert in swallowing re-education, a physiotherapist, and a speech therapist was performed.

## 4. Statistical Analysis

All analyses were performed using Stata 16 (StataCorp, College Station, TX, USA). Data were described with the mean, standard deviation (SD), median and 25th–75th percentiles if continuous and as counts and percent if categorical. Non-parametric correlations between continuous variables were assessed with the Spearman R test. The association of categorical variables was assessed by means of Fisher’s exact test. A *p*-value < 0.05 was considered statistically significant.

## 5. Results

The TGJ was placed at a mean age of 8.6 ± 5.6 months. The procedure was uneventful and well-tolerated in all seven cases.

By 6.6 ± 2.0 months after TGJ placement, all patients had undergone GERD surgery. The procedures were performed smoothly with no intraoperative complications. The gastrostomy was removed after 4–6 weeks once oral feeding was successfully established. As indicated in Table 2, six out of seven patients developed anastomotic stenosis that was successfully managed by serial endoscopic dilatations.

### 5.1. Growth and Nutritional Aspects

At the time of TGJ insertion, we recorded a mean weight of 5.8 ± 1.1 kg (z-score −2.68 ± 0.8). The mean weight at fundoplication was 8.9 ± 1.1 kg (z-score −0.9 ± 0.2), with a significant increment compared to the TGJ stage (*p* < 0.001). The clinical parameters during surgical stages are reported in Table 2.

Enteral nutrition started soon after TGJ placement with continuous administration of monomeric solutions. The final full regimen with two boluses per day and continuous night feeding was reached within about 2 weeks, depending on the patient’s tolerance. The length of hospital stay was 2 days.

### 5.2. Post-Surgical Monitoring

A significant difference in hospital admissions between 3 months pre- and post- TGJ insertion was noted (4.8 ± 0.75 vs. 1.6± 0.52, *p* < 0.01). Before treatment, the patients were commonly admitted for respiratory infections, regurgitation, abdominal pain, and stoma care issues; in the 3 months following TGJ, admissions were mainly related to stoma care/tube malfunctioning. In particular, tube dislodgement occurred once in one patient and required endoscopic repositioning, stoma care was required in one patient with recurrent leaks (after the first hospitalization, management of the issue was performed at our outpatient clinic), and one patient had a tube malfunction (he required tube replacement over a guidewire with no need for anesthesia). The remaining four patients had no problems with the TGJ tube (Table 4).

### 5.3. Parental Quality of Life

**Figure 1:**
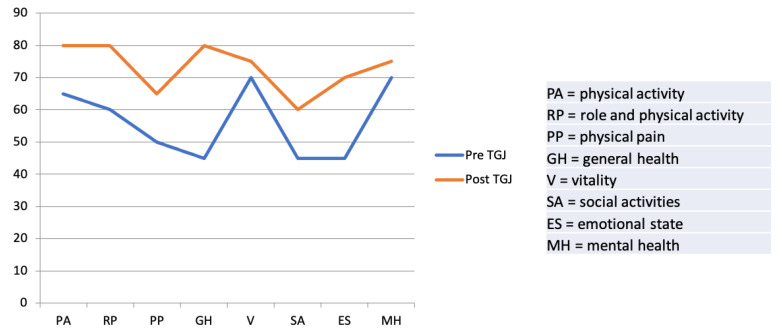
Results from Short-Form 36 questionnaire version 1.6: mean value of each item.

The results are summarized in Figure 1.

We recorded a significant improvement in QoL after TGJ placement, as recorded by higher scores in all 8 items considered. In particular, the biggest improvements were related to parents’ perception of the general health (GH) and emotional state (ES) of their babies, whose scores increased by 35 and 25 points respectively (from 45 to 80 for GH and from 45 to 70 for ES, *p* < 0.01). Physical activity (PA) and pain (PP) improved by 15 points but while PA reached high levels of satisfaction (65 vs. 80 after TGJ placement), PP remained a limiting factor for families (mean score of 50 and 65, respectively before and after TGJ placement, *p* < 0.01). Likewise, social activities, which were very limited before TGJ placement (45 points), improved, reaching a mean score of 60 (*p* < 0.01). Vitality (V) and mental health (MH) were quite satisfactory before TGJ placement, but subsequently improved slightly (score of 70 vs. 75 *p* < 0.01).

## 6. Discussion

Long gap esophageal atresia (EA) (gap ≥ 3 vertebral bodies) occurs in approximately 10% of all infants with esophageal atresia and surgical repair is often difficult with significant postoperative complications. Classical management of patients with LGEA includes the placement of a gastrostomy tube for bolus feed in the neonatal period followed by delayed esophageal anastomosis [8]. In recent years, thoracoscopic treatment of LGEA (thoracoscopic traction technique and anastomosis) has been proposed as an approach that could reduce the prolonged need for a gastrostomy [1,10,11]. Although tempting, this approach is not easily performed. Moreover, the population of LGEA patients have been shown to have multiple comorbidities (e.g., low gestational age, low birth weight) with greater potential for complications and higher perioperative mortality [11,12,13].

Nutritional problems have been commonly reported after restoring esophageal continuity due to altered esophageal anatomy and motility (predisposition to GERD), as well as a delay in initiating oral intake, and parental anxiety [7,12]. Moreover, the reduced neurodevelopmental and social interaction within the first 24 months influences the child’s feeding capability [12,13], and nutritional goals are reached belatedly [12,13,14]. Other influencing factors include the brachy-esophagus, the intrathoracic gastro-esophageal junction, and the type of intervention. [12,15]

In terms of functional aspects of the esophagus, the function of the lower esophageal sphincter (LES) and the motility propagation is often altered by upward traction [12,15,16], complicating the feeding path and predisposing infants to GERD.

Indeed, GERD is a frequent occurrence after delayed repair of LGEA and, together with esophageal dysmotility, exposes patients to aspiration-induced airway infections, nutritional and global impairment, and affects the post-operative course (e.g., GERD contributes to anastomotic strictures, as attested by their high rate in this series).

Different nutritional strategies have been proposed to overcome the above-mentioned problems such as parenteral nutrition, nasogastric tubes, and gastrostomy devices. In particular, gastrostomy feeding enables easy and effective nutritional intake and avoids some of the negative consequences of the other approaches [17]. It also allows us to perform oral stimulations and early oral rehabilitation [18].

Gastrostomy feeding may not be tolerated in the case of GERD-related symptoms (vomiting, regurgitation, malnutrition, respiratory symptoms, recurrent esophageal strictures) and brachy-esophagus [19,20,21].

In these patients, the placement of a TGJ tube is an efficient and safe solution. TGJ also permits gastric decompression using the dedicated port, thus reducing the risk of severe complications of GERD. However, the placement of a jejunal feeding tube should not be considered for widespread use; our experience over the last 10 years has shown that only a limited number of patients require this approach. TGJ appeared to be a possibility in those patients with a pre-existing gastrostomy and associated problems including intolerance to enteral feeding, moderate/severe undernutrition, diagnosis of GERD (confirmed, not just suspected), and symptomatic GERD (recurrent symptoms, including respiratory ones) due to intrathoracic EGJ co-morbidities (e.g., syndromes, heart diseases), that may contraindicate long surgical procedures in the short term. Our series of patients also presented a high rate of prematurity and low birth rates, which are major factors of post-operative complications in the first months of life [22,23].

Each time these factors are identified, the multidisciplinary team caring for the patient should meet and discuss the clinical case in order to make a suitable decision.

The concept of post-pyloric feeding by jejunal feeding tube (JFT) has been well described in children [24,25,26,27]. The ESPGHAN-NASPGHAN guidelines for gastrointestinal complications of EA include the long-term dependency on post-pyloric feeding as an indication for fundoplication (statement 8C) [28].

This approach is less invasive than other anti-reflux procedures and it is generally considered a temporary nutritional strategy to limit long-term parenteral nutrition, when the use of gastrostomy is not possible [27,29,30,31].

However, long-term outcomes and complications of JFT are unclear and reported complication rates vary widely, 1.6–20% [27,30,31]. Most complications are related to tube insertion, maintenance, and mechanical problems [24,29,30,31], as described by Michaud et al. [24]. We did not experience any difficulties in inserting the jejunal tube and we had three mechanical complications (tube dislodgement, obstruction, and leaking), with just one patient requiring general anesthesia to replace the tube. The careful selection of patients, reduced usage time, parental tutoring, and nursing support at our outpatient clinic helped us to optimize the procedure and reduce the complication rate.

Indeed, in our hospital, there is a dedicated team of endoscopists (pediatric surgeons and pediatricians) who routinely perform this type of procedure. The jejunal tube is carefully selected on the basis of the child’s age, length, and weight. This is important not only to facilitate the endoscopic placement of JFT but also to reduce complications such as dislodgement and obstruction. Moreover, we can avail of pediatric and neonatal endoscopes: the use of a 5 mm endoscope was fundamental in smaller babies.

We are also aware of the possibility of cramping, bloating, and dumping [18], but a well-planned and tailored nutrition program (e.g., iso-osmolar formula and infusion according to patients’ tolerance) will help in reducing such complications.

We had positive results in managing seven patients with TGJ in terms of weight gain, reduced hospitalization, and high parental satisfaction. Regarding QoL, the nutrition plan based on two meals during the day and continuous night feeding was particularly welcomed by parents: it somehow reproduces the feeding mode of the family, avoiding the dependence involved in a continuous 24-hour nutrition regimen. We admit that this nutrition plan, specifically the continuous night feeding, is not optimal, but it may be used for a limited period to achieve specific nutritional goals. The number of hospitalizations was also significantly reduced, starting from the first weeks after TGJ placement. TGJ can successfully bridge the gap while waiting to perform a definitive surgical procedure to correct GER. Our data suggest that JPEG positioning could be performed as early as the infant’s first months when growth chart deflection appears. Indeed, fundoplication is essential in these patients and it is required in almost all cases [7].

Some authors reported on the use of early anti-reflux procedures [1,10,11], but we considered it unsafe to perform fundoplication in the presence of severe nutritional deficiencies, complex associated anomalies, short esophagus, and comorbidities. Moreover, we should remind the reader of the well-known problems of esophageal and gastric motility disorders that are frequently observed in EA patients and are important factors predisposing to GER symptoms [32]. We, therefore, decided to postpone surgery until after a period of jejunal enteral feeding and patient stabilization.

We are aware of the limitations of this study, mainly related to the small and heterogeneous sample of patients without a control group, which limits statistical analysis; further studies are mandatory to confirm our results. Moreover, we used only patients’ weight as an indicator of growth, because weight shift is usually the most sensitive variable to growth changes over short monitoring periods. Despite these limitations, this preliminary study indicates how TGJ is not the procedure of choice, but can be a bridge treatment option for nutritional problems in LGEA with a short esophagus.

In conclusion, the placement of the TGJ as a temporary nutritional tool in selected cases of LGEA, with intrathoracic GEJ and growth failure, could improve nutritional conditions and parental QoL in the period before fundoplication, enabling successful surgical correction of GERD.

## Figures and Tables

**Table 1:** Patients’ data and esophageal atresia (EA) type.

Patient	Sex	Gestational Age (Term/Preterm)	Birth Weight, kg (Z-Score) ^1^	EA Type	Associated Anomalies
1	M	Term	3.1 (−0.77)	A	Aberrant right subclavian artery; severe tracheomalacia
2	M	Term	2.9 (−1.23)	A	Cardiac anomaly
3	F	Preterm *	1.3 (−2.22)	A	Cardiac anomaly
4	M	Preterm	2.1 (−1.01)	C	IVs adrenal neuroblastoma
5	F	Preterm	1.4 (−1.37)	C	ARM, right-sided aorta
6	F	Preterm	1.5 (−1.85)	A	VACTER; palatoschisis
7	M	Term	3.2 (−0.55)	A	Tracheomalacia; congenital airways malformation

* + prenatal amnio decompression. ^1^ z-score from WHO and 2013 Fenton growth charts for preterm infants.

**Table 2:** Type of esophageal surgery, post-surgical complications and location of esophago-gastric junction (EGJ).

Patient	Type of EA	Long Gap (Vertebral Bodies)	Type of Anastomosis	EGJ Position after Esophageal Anastomosis	EGJ Position after Fundoplication	Post-Surgical Complications
1	A	5	-Thoracic elongation -Delayed primary anastomosis	3 vertebral bodies above the diaphragm	N/A	Anastomotic stenosis; GER; intrathoracic EGJ; short esophagus
2	A	4	-Thoracic elongation -Delayed primary anastomosis	3 vertebral bodies above the diaphragm	2.5 intercostal spaces above the diaphragm	Anastomotic stenosis; GER; intrathoracic EGJ; short esophagus
3	A	6	-Jejunal interposition	-	-	Anastomotic stenosis; GER; short esophagus
4	C	4	-Thoracic elongation -Gastric pull-up *	2 vertebral bodies above the diaphragm	1.5 intercostal spaces above the diaphragm	Anastomotic stenosis; GER; intrathoracic EGJ
5	C	3	-Thoracic elongation -Delayed primary anastomosis	2 vertebral bodies above the diaphragm	1.5 vertebral bodies above diaphragm	no
6	A	6	-Gastric transposition *	-	-	Anastomotic stenosis; GER; intrathoracic EGJ; short esophagus
7	C	3	-Gastric pull-up *	2 vertebral bodies above the diaphragm	2 intercostal spaces above the diaphragm	Anastomotic stenosis; GER; intrathoracic EGJ; short esophagus

N/A, not available; GER, gastro-esophageal reflux; EGJ, esophago-gastric junction. * Gastric pull-up indicates mobilization of the lower esophageal pouch together with the gastric fundus; gastric transposition procedure is an esophageal replacement technique carried out for esophageal substitution. In both cases, after traction of the EGJ, we performed an intrathoracic anastomosis.

**Table 3:** Clinical parameters of the patients at different surgical stages.

Patient	Gastrostomy			J-Tube-insertion			Fundoplication		
	Age (days)	Weight (kg)	Weight z-score	Age (months)	Weight (kg)	Weight z-score	Age (months)	Weight (kg)	Weight z-score
1	1	3.1	−0.77	4	5.3	−2.4	4.8	5.5	−2.1
2	2	2.9	−1.23	5.9	5	−2.6	16.5	7.5	−2.3
3	1	1.3	−2.22	2.9	3.7	−2.5	5	5.4	−2.0
4	1	2.1	−1.01	2.8	4.6	−1.5	5.5	5.8	−2.3
5	1	1.4	−1.37	3.8	3.7	−3.5	4.8	3.8	−4.2
6	1	1.5	−1.85	10.9	5.3	−4.0	17	6.5	−3.7
7	1	3.2	−0.55	2.1	5	−0.9	6.3	6.2	−2.2

**Table 4:** Number (n) of admissions pre- and post-transgastric jejunostomy (TGJ) placement and complications related to TGJ.

Patient	Admissions pre TGJ (n)	Admissions Post TGJ (n)	TGJ complications
1	4	2	None
2	5	1	None
3	4	2	Tube dislodgement (endoscopic repositioning)
4	6	2	Stoma care
5	5	1	None
6	5	1	None
7	5	2	Tube malfunction (replacement without anesthesia)

## Data Availability

Data is contained within the article.
